# A Human iPSC-Derived Tri-Culture Model of NeuroHIV: HIV Infection, Antiretroviral Drug Effects, Microglial Morphology, and Neuronal Calcium Dynamics

**DOI:** 10.3390/v18101065

**Published:** 2026-09-25

**Authors:** Kara L. Gordon, Christine G. Rines, Laura-Maria Oja, Steve Gilmore, Lilian Harrison, Alyson Smith, Randall Ingermanson, Vez Repunte-Canonigo, Jefferey H. Price, Patrick M. McDonough, Pietro Paolo Sanna, Babu L. Tekwani

**Affiliations:** 1 Vala Sciences, Inc., San Diego, CA 92121, USA; crines@valasciences.com (C.G.R.); loja@valasciences.com (L.-M.O.); sgilmore@valasciences.com (S.G.); lharrison@valasciences.com (L.H.); asmith@valasciences.com (A.S.); ringermanson@valasciences.com (R.I.); jprice@valasciences.com (J.H.P.); pmcdonough@valasciences.com (P.M.M.); 2 Department of Immunology and Microbiology, The Scripps Research Institute, La Jolla, CA 92037, USA; canonigo@scripps.edu

**Keywords:** neuroHIV, iPSC, microglia, antiretroviral therapy, neuronal calcium, morphology, tri-culture, dolutegravir, bictegravir, neurotoxicity

## Abstract

HIV-associated neurocognitive disorders (HAND) can persist despite effective ART and are associated with persistent central nervous system (CNS) viral reservoirs in microglia, chronic neuroinflammation, synaptic dysfunction, and neuronal injury. Thus, human-relevant multicellular CNS models are needed to elucidate neuroHIV pathogenesis and ART neurotoxicity. Here, we establish and characterize a fully human induced pluripotent stem cell (hiPSC)-derived CNS tri-culture model comprising isogenic neurons, astrocytes, and microglia that remains viable and functionally active for up to 32 days in vitro. Using a microglia-tropic fluorescent HIV reporter virus, GFP-HIV-AD8, we demonstrate infection of IBA1+ microglia with no detectable infection of IBA1− cells or TUBB3+ neurons. HIV replication was quantified over 14 days post-infection and effectively suppressed by the first-generation integrase strand transfer inhibitor (INSTI)-containing regimen elvitegravir/tenofovir disoproxil fumarate/emtricitabine (EVG/TDF/FTC), and the second-generation INSTI-containing regimens dolutegravir/tenofovir disoproxil fumarate/emtricitabine (DTG/TDF/FTC) and bictegravir/tenofovir alafenamide/emtricitabine (BIC/TAF/FTC), at concentrations based on reported plasma Cmax values. HIV infection reduced microglial viability by approximately 70%, partially rescued by lower in vitro ART concentrations but worsened by the supratherapeutic 3× Cmax condition of DTG-containing regimens. In uninfected cultures, DTG induced microglial morphological changes consistent with a reactive or dysfunctional state. Calcium imaging revealed ART-specific neuronal effects: EVG/TDF/FTC reduced neuronal activity, whereas BIC/TAF/FTC enhanced it. In HIV-infected cultures, DTG shortened neuronal spike duration under viral suppression, identifying an HIV–DTG-associated electrophysiological phenotype. Overall, BIC/TAF/FTC showed the most favorable profile across the cellular and electrophysiological endpoints assessed in this donor line. Together, these findings establish an hiPSC-derived CNS tri-culture model for controlled studies of neuroHIV virology, ART effects, and neuronal function.

## 1. Introduction

HIV-associated neurocognitive disorders (HAND) continue to affect 30–50% of people living with HIV despite effective viral suppression on combination antiretroviral therapy (ART) [1,2]. The prevalence of HAND is actually increasing in the aging HIV population, with milder forms such as asymptomatic neurocognitive impairment and mild neurocognitive disorder dominating the clinical spectrum [3,4]. The pathogenesis of HAND is multifactorial, involving expression of HIV proteins and persistent CNS viral reservoirs, chronic immune activation, neuroinflammation, and ART-related neurotoxicity [5,6,7,8,9,10,11,12,13]. Microglia, the resident myeloid cells of the CNS, are an established cellular reservoir for HIV in the brain and can harbor latent or low-level replicating virus even in the setting of suppressive ART [6,14]. Infected microglia secrete neurotoxic factors, including inflammatory cytokines, chemokines, and viral proteins such as gp120 and Tat, which indirectly disrupt neuronal function and survival [2,15,16]. Direct infection of neurons does not occur, a feature our model recapitulates.

HIV infection of humanized mouse models and HIV-1 transgenic rodents, and SIV infection of macaques, have provided critical insights into neuroHIV pathogenesis [17,18,19]. However, they are costly, low-throughput, and often fail to fully replicate human-specific viral–host interactions, neuroimmune signaling, or ART pharmacokinetics [18,20,21]. Immortalized cell lines, such as U937 microglia or SH-SY5Y neurons, lack primary cell characteristics and do not support the complex neuron–astrocyte–microglia cross-talk essential for CNS homeostasis and pathology [22,23]. Primary human fetal or adult CNS cells are scarce, variable, and ethically challenging to obtain.

The National NeuroAIDS Tissue Consortium (NNTC) provides clinically annotated antemortem data, biofluids, and postmortem CNS tissues from PWH and PWoH [24]. These resources provide direct human tissue context and complement live experimental models. Postmortem material is cross-sectional/endpoint and cannot provide the same live, longitudinal and functional readouts, including real-time viral kinetics and dynamic calcium imaging, as the tri-culture.

In recent years, human-induced pluripotent stem cell (hiPSC) technology has revolutionized disease modeling [22]. iPSC-derived neurons, astrocytes, and microglia can be generated in large quantities from individual donors, enabling patient-specific modeling of neuroHIV [25]. However, there is a need for iPSC-based CNS models containing multiple disease-relevant cell types that replicate HIV infection dynamics and support studies of neuroHIV virology, ART efficacy testing, and neurotoxicity assessment. Most existing platforms are either monocultures or simple dual co-cultures (e.g., neuron–astrocyte), lacking the full tri-cellular architecture that includes microglia, the primary target of HIV in the brain.

Here, we established an hiPSC-derived tri-culture model containing neurons, astrocytes, and microglia, which is amenable to high-content imaging and functional readouts such as dynamic single-cell calcium imaging, and tested HIV infection and the effects of ART combinations containing integrase strand transfer inhibitors (INSTIs). The INSTIs block the strand-transfer step of HIV integration into the host genome and are currently a preferred first-line ART worldwide [26]. Five INSTIs have been introduced to date [26]. The first-generation INSTI elvitegravir (EVG) remains clinically available in fixed-dose combinations [26]. The second-generation INSTIs dolutegravir (DTG) and bictegravir (BIC) are currently more widely used as first-line antiretroviral therapy due to their higher genetic barriers to resistance [26].

We show that the hiPSC-derived tri-culture containing neurons, astrocytes, and microglia remains healthy for up to 32 days in vitro (DIV32) and demonstrate that: (1) this model supports productive HIV infection in IBA1+ microglia; (2) HIV replication can be quantified longitudinally via GFP expression; (3) three major ART combinations suppressed HIV replication effectively; (4) HIV infection reduced microglial viability, with partial rescue at lower in vitro ART concentrations but exacerbation at the supratherapeutic 3× Cmax DTG/TDF/FTC condition; (5) DTG and DTG/TDF/FTC at 3× Cmax induced microglial morphological changes consistent with activation or toxicity; (6) ART combinations showed differential effects on neuronal calcium dynamics, with second-generation INSTI combinations, and BIC/TAF/FTC in particular, showing a favorable profile; and (7) HIV infection in the presence of DTG shortened neuronal spike duration, a novel finding with implications for synaptic dysfunction in HAND.

This model provides a controlled, quantitative system for studies of HIV replication in human CNS-derived cells, ART effects, and neuron–glia interactions, with a focus on infected microglia and neuronal function. It is framed as a tractable, standardized in vitro system for comparative drug testing, hazard identification, and mechanistic hypothesis generation, particularly for translational neuroscientists and virologists. These findings are particularly relevant to the FDA’s emphasis on New Approach Methodologies (NAMs), including human in vitro models, to improve translational predictivity, reduce reliance on animal testing, and support more efficient drug development.

## 2. Materials and Methods

### 2.1. Human iPSC (hiPSC)-Derived CNS Tri-Culture Model

Isogenic hiPSC-derived neurons, astrocytes, and microglia were obtained from BrainXell (Madison, WI, USA). All three cell types were generated using BrainXell proprietary differentiation protocols. Commercial sourcing was a deliberate design choice to prioritize lot-to-lot reproducibility, quality control, and inter-laboratory transferability. For tri-culture assembly in HIV experiments (day in vitro 0, DIV0), cortical glutamatergic neurons and cortical astrocytes were thawed and seeded into 96-well plates (Revvity, Waltham, MA, USA, #6055302) pre-coated with Poly-D-Lysine (Sigma, St. Louis, MO, USA, P0899) at a final density of 27,200 neurons and 3400 astrocytes (neuron:astrocyte ratio 8:1) in BrainXell recommended co-culture media containing Geltrex (Gibco, Waltham, MA, USA, A14132-01). Co-cultures were maintained according to BrainXell’s recommendations. On DIV7 or DIV9, 13,600 microglia were added per well (neuron:microglia ratio of 8:4). Tri-cultures were maintained in BrainXell’s recommended media Phenol Free Neurobasal (Gibco 12348-017) and Phenol Free DMEM/F12 (Gibco 11039-021) supplemented with B27 (Gibco 17504-044), N2 (Defined Biosciences, San Diego, CA, USA, HiDef N2), GlutaMAX (Gibco 35050-061), ascorbic acid (Thermo Fisher, Waltham, MA, USA, 036237.14), Chemically Defined Lipid Mix (Gibco 11905-031), 20 ng/mL M-CSF (Peprotech, Cranbury, NJ, USA, 300-25), 100 ng/mL IL-34 (Peprotech 200-34). Half-medium changes were performed every 3–4 days. For tri-culture assembly for ARV toxicity studies, co-cultures in 384-well plates pre-coated with Poly-D-Lysine were plated at neuron:astrocyte ratio 4:1 (8800 neurons and 2200 astrocytes per well). On DIV9 microglia were stained with Cellvue Far Red membrane dye (Sigma, MINCLARET-1KT) and were added per well (neuron:microglia ratio of 5:1; 1760 microglia per well). Cultures were used for experiments at DIV21–32.

### 2.2. HIV-1 Strain and Infection

The GFP-expressing HIV-1 strain GFP-HIV-AD8 (HIV-1(AD8)-GFP) was custom ordered from Virongy Biosciences (Manassas, VA, USA). This strain is based on the pNL-AD8 molecular clone (CCR5-tropic) that has been modified so the GFP is produced on the same reading frame as the NEF gene; the NEF gene is expressed from an internal ribosome entry site (IRES) downstream of GFP. The stock titer was approximately 1.46 × 10^6^ IU/mL.

For infections, tri-cultures at DIV11 or DIV12 were exposed to virus diluted in culture medium at 1:250 final dilution (corresponding to approximately 5840 IU per well) for 24 h at 37 °C in 5% CO_2_. Following infection, the medium was discarded, cultures were washed once with complete media and replaced with fresh complete medium. Uninfected controls were handled identically using complete media only (no virus). A heat-inactivated or replication-incompetent virus control was not included; therefore, the present study does not distinguish effects of productive replication from effects of viral-particle or envelope exposure in the absence of replication. GFP expression (indicating HIV replication) was monitored daily for 14–28 days post-infection (DPI) using Vala Sciences’ Kinetic Image Cytometer (IC200 KIC^®^) (San Diego, CA, USA).

### 2.3. Antiretroviral Drugs

The following antiretroviral drugs were obtained from commercial sources: Dolutegravir (DTG; HY-13238), elvitegravir (EVG; HY-14740), bictegravir (BIC; HY-17605), tenofovir disoproxil fumarate (TDF; HY-13782) and tenofovir alafenamide (TAF; HY-15232) all from MedChemExpress (Monmouth Junction, NJ, USA), emtricitabine (FTC; E525000) from Toronto Research Chemicals (Vaughan, ON, Canada). All drugs were reconstituted in DMSO (Sigma, D2650) as 100 mM stock solutions and stored at −80 °C. Working dilutions were prepared fresh in culture medium on the day of use. The final DMSO concentration never exceeded 0.1%, which served as vehicle control.

Concentrations tested were based on published maximum plasma concentration (Cmax) values from clinical studies: DTG: 8 µM; EVG: 3.5 µM; BIC: 13.5 µM; TDF: 0.6 µM; TAF: 0.6 µM; FTC: 7 µM.

Drugs were tested alone or in clinically relevant combinations: DTG/TDF/FTC (integrase inhibitor + NRTI backbone); EVG/TDF/FTC (integrase inhibitor + NRTI backbone); BIC/TAF/FTC (integrase inhibitor + NRTI backbone). Each combination was tested at 0.1×, 1×, and 3× Cmax of each component, as well as a fixed concentration of 10 µM each. For HIV infection studies, antiretrovirals (ARVs) were added starting at DPI 1 (or DPI 4 for delayed treatment experiments) and continued for 7–14 days with twice-weekly half-medium changes to maintain drug levels. For uninfected toxicity studies, ARVs were added between DIV22 and DIV25 for 7 days.

Concentrations up to 3× the reported human plasma Cmax (3× Cmax) were included to provide a conservative supratherapeutic exposure margin and detect subclinical or concentration-dependent neurotoxicity that may not be apparent at standard therapeutic exposure but could emerge in susceptible patient populations or specific dosing scenarios. This approach is consistent with nonclinical safety testing [27]. Additionally, nominal concentrations used in vitro are not directly equivalent to in vivo exposure due to differences in protein binding, and freely available drug concentrations can substantially affect in vitro-to-in vivo extrapolation [28,29]. This is particularly relevant for TDF, TAF, and FTC because plasma parent-drug concentrations do not directly reflect intracellular exposure to their active phosphorylated metabolites [30]. DTG-associated neuropsychiatric adverse events and discontinuations occur more frequently in women and older patients, suggesting greater susceptibility in certain populations [31]. Higher CNS drug exposure may also occur in specific clinical settings because of drug–drug interactions—particularly CYP3A4 inhibition with EVG—altered blood–brain barrier integrity during neuroinflammation, or variability in the expression or activity of efflux transporters such as P-gp and BCRP [32,33,34,35]. Thus, testing up to 3× Cmax provides a conservative hazard-identification condition and helps define the concentration range between antiviral efficacy and measurable cellular toxicity.

### 2.4. Live-Cell Imaging of HIV Replication (GFP Quantification)

HIV replication was monitored longitudinally using GFP expression as a surrogate for p24 expression. Live tri-cultures were imaged daily (5 per week) from DPI 1 to DPI 17 using Vala Sciences’ IC200 Kinetic Image Cytometer (KIC^®^) equipped with a 20× objective (0.75 NA). For each well, a 3 × 3 grid of fields (9 fields total) was acquired. GFP+ microglia were identified using automated image analysis.

### 2.5. Immunofluorescence and High-Content Imaging

At experimental endpoints, cultures were fixed with 2% PFA and 1.7% sucrose in PBS for 15 min at room temperature, washed three times with PBS, and permeabilized with 0.3% Triton X-100 (Sigma, T8787) in PBS for 15 min. Blocking was performed with 5% Goat serum and 1% BSA in PBS for 1 h at room temperature. Primary antibodies (all from commercial sources) were diluted in blocking buffer and incubated overnight at 4 °C: Chicken anti-TUBB3 (neurons; Synaptic Systems, Gottengen, Germany, 302–306; 1:250); Mouse anti-GFAP (astrocytes; Cell Signaling, Danvers, MA, USA, 367S; 1:300); and Rabbit anti-IBA1 (Wako, Richmond, VA, USA, 019-19741; 1:500).After three washes with PBS, secondary antibodies were diluted in 2% BSA in PBS and applied for 1 h at room temperature: Goat anti-Mouse, Alexa Fluor™ 488 (Invitrogen, Waltham, MA, USA, A11029; 1:1000), Goat anti-Chicken, Alexa Fluor™ 555 (Invitrogen, A21437; 1:1000), Goat anti-Rabbit, Alexa Fluor™ 647 (Invitrogen, A21245; 1:1000).

Nuclei were stained with Hoechst 33342 (Invitrogen, H3570; 1:1000).

Plates were imaged using Vala Sciences’ IC200 Kinetic Image Cytometer (KIC^®^) equipped with a 20× objective (0.75 NA). For each well, a 3 × 3 grid of fields (9 fields total) was acquired. Each image was acquired as a Synthetic Focus with a range of 10 µm and step size of 1 µm to ensure sharp focus across the entire well.

### 2.6. Calcium Imaging (Neuronal Activity)

Neuronal activity was assessed using the fluorescent calcium indicator Cal-520 AM (AAT Bioquest, Pleasanton, CA, USA) or Cal-590 AM (AAT Bioquest). At the end of ART treatment (DIV28 for uninfected studies; DPI 17 for HIV-infected studies), culture medium was replaced with Brainphys Phenol Free Imaging Buffer (StemCell Technologies, Vancouver, BC, Canada) containing 5 µM Cal-520 AM (non-infected cultures) or 2.5 µM Cal-590 AM (HIV cultures), 1× Powerload, 1 mM Probenecid, and 0.1 µg/mL Hoechst 33342 (all from ThermoFisher) for 45 min at 37 °C. Cells were then washed twice with Brainphys and allowed to equilibrate for 15 min at 37 °C in the IC200 KIC. Kinetic imaging was performed using the IC200 KIC^®^ at 37 °C with 5% CO_2_ with 1 field of view per well imaged using a 20× objective. A single image of the nuclear channel was obtained, followed by a single image of the CellVue-stained microglia, and the calcium was acquired by imaging four frames per second for 2 min (480 frames, non-infected cultures) or 20 fps for 1 min (infected cultures).

### 2.7. Image Analysis and Quantification (CyteSeer^®^)

All image analysis was performed using Vala Sciences’ CyteSeer^®^ automated image analysis software (v3.0.2.6), which identifies, segments, and indexes each cell. For live HIV-GFP analysis: GFP+ cells were counted per well, and the mean ± SD from N = 6 wells was calculated for each time point. For longitudinal live-cell analysis, GFP+ cells were detected without preselection using a cell-type marker. At experimental endpoints, the cellular identity of GFP+ cells was assessed by immunostaining, including colocalization with IBA1. For fixed cell analysis: cells were first indexed based on Hoechst nuclear staining. Microglia were classified by IBA1-positive (+) nuclei, and infected microglia were classified as IBA1+ and GFP+. Microglia morphology analysis was performed on IBA1+ cells using CyteSeer^®^ algorithms. For each individual cell, the following parameters were calculated: cell area (µm^2^): total area of IBA1 staining; cell body width (µm): average width of the cell body; roundness: = 4 × area/(π × major axis^2^); values close to 1 indicate perfect circularity; elongation: = 1 − (width/length); higher values indicate more elongated cells; for population-level analysis, mean values per well were used. Calcium trace analysis was performed using Vala Sciences’ CyteSeer^®^ AI-enabled image analysis software. Measurements are made on single-cell traces and single transients in each trace using a custom algorithm to baseline the calcium trace (ΔF/F), identify transients, and define the upstroke portion of a calcium transient as an activity spike. For each active neuron, the following parameters were calculated: percent active: percent of neurons with at least one spike; event frequency (spikes per second, Hz); spiking fraction: fraction of time the neuron was spiking; mean spike amplitude (ΔF/F); max rise speed (ΔF/F per second). A neuron was considered “active” if it fired at least one spike during the recording. For each well, data from all cells were aggregated, and well-level means were calculated.

### 2.8. Statistical Analysis

All experiments were performed with N = 4–6 technical replicate wells per condition from one independent experiment using the same cell lot. Data are presented as mean ± standard deviation (SD). Comparisons between two groups were performed using two-tailed Student’s *t*-test (parametric) or Mann–Whitney U test (non-parametric). For multiple comparisons, one-way ANOVA with Dunnett’s post hoc test (comparing all conditions to control) or two-way ANOVA with Šídák’s multiple-comparisons test (for two independent variables, e.g., infection status and treatment) was used. Accordingly, reported *p*-values from multi-group analyses reflect the indicated Dunnett or Sidak multiple-comparison correction. Statistical analyses were performed using GraphPad Prism 9.5 (San Diego, CA, USA). Significance thresholds: * *p* < 0.05, ** *p* < 0.01, *** *p* < 0.001, **** *p* < 0.0001.

## 3. Results

### 3.1. hiPSC Tri-Culture Model Supports Long-Term CNS Cell Survival and Maintains Cell-Type-Specific Marker Expression

We first characterized the baseline health and composition of the tri-culture model at DIV 32, the latest time point evaluated (Figure 1). Immunofluorescence staining revealed clear and spatially organized expression of cell-type-specific markers. TUBB3 (neurons) was present in a substantial proportion of total cells, with elaborate neurite networks extending across the well surface. Neuronal cell bodies appeared phase-bright and formed small clusters, consistent with synaptic network formation. GFAP (astrocytes) was expressed in a large fraction of cells, with a stellate morphology and fine processes interacting with both neurons and microglia. IBA1 (microglia) was present in a smaller subset of cells, displaying a ramified morphology under basal conditions, a small cell body with multiple thin, branching processes, characteristic of surveillant, non-activated microglia (Figure 1A). Hoechst nuclear staining confirmed that total cell density remained stable from DIV 21 to DIV 32, with no evidence of spontaneous cell death or detachment. The overlay image (Figure 1B) demonstrated close physical association between the three cell types, with microglial processes often contacting neuronal somata and dendrites. Importantly, no signs of spontaneous activation or reactive gliosis were observed, as assessed by IBA1 intensity and GFAP upregulation. This model thus provides a stable, long-lived, physiologically relevant CNS microenvironment suitable for HIV infection and drug exposure studies lasting up to 4 weeks.

### 3.2. Detectable HIV Reporter Expression Localizes to Microglia in Tri-Culture

To model HIV infection in the CNS, we exposed tri-cultures at DIV 11 to GFP-HIV-AD8 (1:250 dilution) or uninfected control. At DPI 17, cultures were fixed and stained for TUBB3 (neurons), IBA1 (microglia), and Hoechst (nuclei). GFP expression (HIV) was visualized directly without immunostaining. Across all fields examined (N = 6 wells, 9 fields/well), all detectable GFP+ cells were IBA1+ (Figure 2). In contrast, uninfected cultures showed no GFP signal in any cell type. Quantitative analysis of neuronal (TUBB3+), microglial (IBA1+), and GFP+ populations across 10 random fields per well confirmed that all GFP+ cells were IBA1+, and none were TUBB3+. GFAP staining was not performed in this experiment; therefore, astrocyte infection was not directly assessed. No GFP+/IBA1− cells were observed. The data directly exclude neuronal (TUBB3+) infection but do not independently assess astrocyte identity because GFAP was not co-stained.

### 3.3. Longitudinal Quantification of HIV Replication in Tri-Culture Using GFP Expression

We next tracked the temporal dynamics of HIV replication over 14 days post-infection (DPI) using live-cell GFP imaging. At DPI 1, only rare isolated GFP+ microglia were observed (Figure 3A, bottom row, left panel). By DPI 7, the number of GFP+ microglia increased markedly, and small clusters of infected cells appeared, suggesting local viral spread (Figure 3A, bottom row, middle panel). At DPI 14, GFP+ microglia were widespread throughout the well, with many cells exhibiting bright GFP fluorescence (Figure 3A, bottom row, right panel). Non-infected cultures showed no GFP+ cells at any time point. To confirm that the GFP signal reflected authentic viral replication and not pseudotyping or residual inoculum, we fixed cultures at DPI 14 and immunostained for IBA1. All GFP+ cells were IBA1+, and their morphology ranged from ramified to amoeboid, consistent with HIV-induced microglial activation (Figure 3B). Quantitative time-course analysis (Figure 3C) revealed an increase in GFP+ microglia count, with a lag phase (DPI 1–3), a slow rise phase (DPI 3–9), and an exponential growth phase (DPI 9–14). Two-way ANOVA with Šídák’s multiple-comparisons test showed significant differences between HIV-infected and uninfected controls at all time points from DPI 7 onward (*p* < 0.01 on D7). The intra-assay coefficient of variation across multiple wells was low, indicating excellent reproducibility. This longitudinal GFP readout enables non-invasive, real-time monitoring of HIV replication and can be used to assess antiviral efficacy and infection dynamics.

### 3.4. Clinically Relevant ART Combinations Suppress HIV Replication in Tri-Culture

We next asked whether the tri-culture model could recapitulate the antiviral efficacy of standard ART combinations. Three modern integrase inhibitor-based regimens were tested: DTG/TDF/FTC, EVG/TDF/FTC, and BIC/TAF/FTC. ART was added starting at DPI 4 and maintained for 13 or 17 days, with half-medium changes every 3 days with fresh drug addition to sustain drug levels. GFP+ microglia counts were quantified five times a week for 2+ weeks after infection. In the untreated HIV control, GFP+ microglia increased exponentially as described above (Figure 4A–C, blue curves). In contrast, all three ART combinations at 0.1× Cmax, 1× Cmax, and 3× Cmax nearly completely suppressed HIV replication, with GFP+ counts remaining at baseline levels throughout the 13 or 17-day time course (orange, green, and pink curves). The BIC/TAF/FTC 3× Cmax condition was not included in the comparative toxicity analysis because the culture was terminated before post-fix quantitative assessment could be performed; therefore, no conclusion regarding drug-specific toxicity was drawn from this condition. No significant differences in antiviral efficacy were observed between the three ART combinations at matched concentration multiples. Notably, the BIC/TAF/FTC combination appeared to suppress viral replication slightly faster than the DTG- or EVG-based regimens, as divergence from the HIV control begins on DPI 7 and becomes significant on DPI 10 in BIC/TAF/FTC, but divergence does not begin until DPI 10 and significant changes do not appear until DPI 12 and 11, respectively, in the DTG- and EVG-based regimens. These data demonstrate that the tri-culture model is sensitive to ART-mediated suppression and can distinguish between suboptimal and optimal drug concentrations.

### 3.5. HIV Infection Reduces Microglial Viability; ART Effects Are Concentration-Dependent

While ART suppresses viral replication, its impact on microglial survival in the context of HIV infection is not well understood. To address this, we quantified total microglia (IBA1+ nuclei) at DPI 17. HIV infection alone significantly reduced microglial count compared to uninfected control (Figure 5C). Treatment with 0.1× Cmax of DTG/TDF/FTC partially but significantly rescued microglial numbers, suggesting that even suboptimal ART reduces viral cytopathic effects. Similarly, 0.1× Cmax EVG/TDF/FTC also rescued microglia. However, 3× Cmax DTG/TDF/FTC unexpectedly reduced microglial counts below those seen with HIV alone, demonstrating an adverse in vitro effect at this high nominal exposure. In contrast, 3× Cmax EVG/TDF/FTC did not further reduce microglia compared to HIV alone. Notably, neither HIV nor any ART combination significantly altered the total number of neurons (TUBB3+) and astrocytes (GFAP+) (Figure 5B), indicating that the observed effects are microglia-specific. These findings highlight a dual role for ART: protective at lower in vitro concentrations via viral suppression, but potentially harmful at higher in vitro concentrations, particularly with DTG-containing regimens.

### 3.6. ART Alters Microglia Morphology in Uninfected Tri-Cultures

Given the microglial count changes induced by ART, we next examined whether ART alone (in the absence of HIV) affects microglial morphology, a sensitive indicator of activation state. Uninfected tri-cultures were treated with DTG alone or DTG/TDF/FTC at 0.1×, 1×, and 3× Cmax for 7 days. At 3× Cmax, DTG alone significantly reduced the number of live microglia (Figure 6A) and caused striking morphological changes (Figure 7A). IBA1+ cells became more rounded, with fewer processes, and increased cell body width. The DTG/TDF/FTC combination produced even more pronounced effects at 1× and 3× Cmax. Quantitative analysis (Figure 6) showed that DTG alone increased microglial roundness at 3× Cmax and increased cell body width. The combination ART increased roundness and width at both 1× and 3× Cmax. These morphological changes are consistent with a transition from a surveillant, ramified phenotype to an activated, amoeboid-like state, which may have functional consequences for neuroimmune interactions. No significant morphological changes were observed with EVG or BIC combinations at any concentration, suggesting that DTG is a primary driver of microglial morphological alteration.

### 3.7. HIV Infection Overrides ART Effects on Microglia Morphology

We next asked whether HIV infection itself drives microglial morphology changes and whether ART modifies this effect. In HIV-infected tri-cultures treated with various ART regimens, we separately analyzed GFP+ (infected) and GFP– (uninfected bystander) microglia within the same well. Strikingly, regardless of ART treatment, GFP+ microglia were significantly more elongated and less round compared to GFP- microglia (Figure 8B,C). This HIV-induced morphological shift was consistent across all ART conditions, including untreated HIV and fully suppressed (1× Cmax ART) cultures. In contrast, ART alone (in GFP- cells) had no significant effect on elongation or roundness compared to uninfected controls. These data indicate that HIV infection itself is the dominant driver of microglial morphological changes, overriding any modulatory effects of ART. The persistence of morphological alterations even under fully suppressive ART suggests that either low-level residual viral protein expression or irreversible cellular remodeling occurs.

### 3.8. ART Combinations Differentially Affect Neuronal Calcium Activity

To characterize direct neuronal effects of ART under the tested in vitro exposure conditions, we performed calcium imaging to assess network-level neuronal activity after 7-day ART exposure in uninfected tri-cultures. EVG/TDF/FTC at 3× Cmax significantly reduced the number of active neurons per field, event frequency, spiking fraction, mean spike amplitude, and max rise speed (Figure 9B, middle panels). DTG/TDF/FTC at 3× Cmax also reduced the percent of active neurons and event frequency, with only trends toward reduction of other parameters (Figure 9A, top panels). BIC/TAF/FTC showed the most favorable profile: at 0.1× Cmax and 10 µM, it increased event frequency and spiking fraction, and at 10 µM increased mean spike amplitude with no reductions in calcium activity at any dose (Figure 9, bottom panels). These data characterize distinct ART-associated neuronal activity profiles in this in vitro system.

### 3.9. HIV Infection Combined with DTG Shortens Neuronal Spike Duration

Finally, we examined whether HIV infection modifies ART effects on neurons. HIV-infected tri-cultures were treated with DTG (Cmax, 8 µM) added either at DPI 1 (early) or DPI 4 (delayed). Both treatment regimens fully suppressed viral replication by DPI 10 (Figure 10A,B). Calcium imaging at DPI 14 revealed that in HIV-infected cultures treated with DTG (DPI 1), neuronal spike duration was significantly shorter compared to uninfected cultures treated with DTG (Figure 10C). No differences were observed in spike frequency, amplitude, or rise speed. The DPI 4 treatment group showed a similar trend but did not reach statistical significance. These results identify an interaction between HIV infection and DTG exposure on neuronal spike duration under conditions of viral suppression.

## 4. Discussion

We have developed and characterized a hiPSC-derived tri-culture model of neuroHIV that reproduces selected aspects of CNS HIV infection and ART exposure. The model provides several experimental features: all three cell types are derived from isogenic hiPSCs, avoiding species and genetic background differences; the cultures remain stable for >4 weeks, enabling long-term studies; detectable HIV reporter expression localizes to microglia under the conditions tested; viral replication can be tracked in real time via GFP; the system is compatible with high-content imaging and functional calcium readouts; and it is compatible with 96- or 384-well formats for quantitative studies. Below, we discuss the implications of our key findings.

The principal novelty of the present study lies in the integration of commercially sourced, isogenic neuron–astrocyte–microglia components with longitudinal, cell-resolved virologic, morphologic, pharmacologic, and advanced functional readouts. Commercial sourcing was a deliberate design choice to prioritize lot-to-lot reproducibility, quality control, and inter-laboratory transferability. All three lineages are derived from a single hiPSC donor, which holds genetic background constant across cell types and treatment conditions and helps isolate infection- and treatment-related effects, while not addressing interindividual variability. The cultures remain viable and imageable through DIV32. GFP-HIV-AD8 enables longitudinal quantification of infection in microglia. Within-well comparison of GFP+-infected and GFP− bystander microglia permits direct assessment of infection-associated morphology under identical culture and treatment conditions. The present experiments were designed to model productive microglial infection and subsequent pharmacologic suppression, not HIV latency or reservoir reactivation.

### 4.1. Selective Microglial Infection and Viral Spread

Our observation that all detectable GFP+ cells were IBA1+ and none were TUBB3+ is consistent with the known restricted tropism of HIV for CD4+ myeloid cells in the CNS [11,12,13]. GFAP staining was not performed in the infection experiment; therefore, the data directly exclude neuronal (TUBB3+) infection but do not formally exclude astrocyte infection. Astrocytes have been reported to contain integrated HIV DNA and, although relatively non-permissive to cell-free HIV entry and lacking canonical CD4, to support low-level, non-cytopathic, CD4-independent infection [36,37]. Despite poor infectivity and replication, efficient cell-to-cell viral transfer has been reported [37]. We focused on microglia in this model because the potential contribution of astrocytes to productive infection may happen through non-classical mechanisms, may represent relatively rare events, and is characterized by limited replication capacity [38,39]. In contrast, microglia and macrophages are established major HIV targets in the brain [11,12,13,40,41].

### 4.2. ART Efficacy and Microglial Toxicity

All three ART combinations suppressed HIV replication effectively, demonstrating the model’s utility for antiviral testing. However, we observed concentration-dependent adverse microglial effects of DTG-containing regimens in vitro. At 3× Cmax, DTG/TDF/FTC reduced microglial counts below those seen with HIV alone. This supratherapeutic condition was included as a hazard-identification and safety-margin exposure and should not be interpreted as representative of standard therapeutic dosing. The tri-culture does not model blood–brain barrier drug penetration, and nominal in vitro concentrations do not directly correspond to CNS drug exposure because protein binding and intracellular drug disposition differ between culture and in vivo conditions. While DTG is generally well tolerated, it has been associated with neuropsychiatric adverse events, including insomnia, anxiety, and depression [42,43,44]. Previous studies suggested that DTG can perturb mitochondrial respiration and immunometabolic function in microglia cell lines, including murine BV2 microglial cells [45] and human microglia-derived hµglia cells [46], although not in hiPSC-derived microglia cells, as we report here.

Our morphological data support DTG-induced activation of hiPSC-derived microglia, as evidenced by increased roundness and reduced ramification, a phenotype associated with enhanced production and release of pro-inflammatory cytokines [47,48]. DTG-induced morphological alterations have also been observed in a microglia cell line and in primary rat oligodendrocytes [45,49]. These findings identify concentration-dependent DTG-associated microglial effects in this in vitro system.

### 4.3. ART-Induced Neuronal Effects

The differential effects of ART combinations on neuronal calcium activity characterize distinct ART-associated neuronal activity profiles in this in vitro system. EVG broadly reduced several neuronal calcium-activity parameters in this in vitro system, including spike frequency and amplitude. EVG has also been shown to alter spontaneous electrical activity in hiPSC-derived neurons, as measured by microelectrode array [50]. BIC/TAF/FTC increased several neuronal calcium-activity parameters in this in vitro system; the physiological and clinical significance of these changes remains to be established.

### 4.4. Additive Effects of HIV and ART on Neuronal Dysfunction

A novel biological finding enabled by the model is that the combination of HIV infection and DTG shortened neuronal spike duration, an effect not observed with either condition alone. Action-potential duration is an important determinant of presynaptic calcium influx and neurotransmitter release and can thereby influence synaptic strength and plasticity [51,52]. Shortened spikes can reduce presynaptic calcium influx and diminish neurotransmitter release, potentially impairing network synchrony and information processing. The mechanistic basis remains to be determined; HIV-associated inflammatory cytokines, including TNF-α and IL-1β, which can modify neuronal sodium and potassium currents, and direct effects of DTG on neuronal physiology are candidate contributors [53,54]. Importantly, this effect occurred despite complete suppression of viral replication, consistent with evidence that neuroinflammation and HIV-associated CNS pathology can persist during suppressive ART [55]. HAND can persist despite effective viral suppression [1,2]; accordingly, this finding identifies a HAND-relevant neuronal phenotype that emerges despite suppression of productive viral replication and warrants future mechanistic investigation.

The current tri-culture does not include oligodendrocytes. The current findings provide a foundation for several important future applications. Future studies will expand this isogenic tri-culture model by incorporating additional CNS cell types, including oligodendrocytes, as well as neurovascular components such as pericytes and endothelial cells, to establish a functional blood–brain barrier [56,57]. The model can also be adapted to microfluidic systems with dynamic flow conditions to more closely model physiological drug transport and distribution across the blood–brain barrier.

The model also provides a basis for testing diverse HIV strains, including patient-derived isolates, to improve translational relevance. The calcium-imaging findings can be extended using complementary electrophysiological approaches, including patch-clamp and multielectrode-array recordings, to characterize changes in neuronal excitability, network activity, and synaptic transmission more directly. Studies using matched hiPSC-derived neurons, astrocytes, and microglia from multiple donor backgrounds will permit controlled interindividual comparisons while maintaining an isogenic background within each tri-culture. Additional viral strains will permit assessment of viral-strain variability.

### 4.5. Limitations

The present study used a single donor-derived isogenic tri-culture and one HIV reporter strain and did not assess donor-to-donor, differentiation-to-differentiation, or viral-strain variability. The model does not reproduce the full human brain microenvironment, systemic immunity, blood–brain barrier, oligodendrocytes/myelin, the years-to-decades clinical timescale, or common comorbidities such as aging and substance use. Detectable HIV reporter expression was observed exclusively in IBA1+ microglia; however, since GFAP staining was not performed in the infection experiment, low-level astrocyte infection cannot be excluded. An inactivated-virus control was not included, so effects of viral-particle exposure independent of active infection were not assessed.

## 5. Conclusions

The hiPSC-derived tri-culture is an experimental model for controlled neuroHIV studies. It enables simultaneous assessment of HIV replication, ART efficacy, microglial health, and neuronal function. Our data reveal differences between ART combinations across the endpoints assessed in this donor line. The model supports controlled studies of productive HIV infection, ART effects, and neuron–glia interactions, including future investigations of how HIV-infected microglia affect surrounding CNS cells. Wider use as a tractable foundation for comparative drug screening and mechanistic hypothesis generation will require validation across additional donors and viral strains.

## Figures and Tables

**Figure 1:**
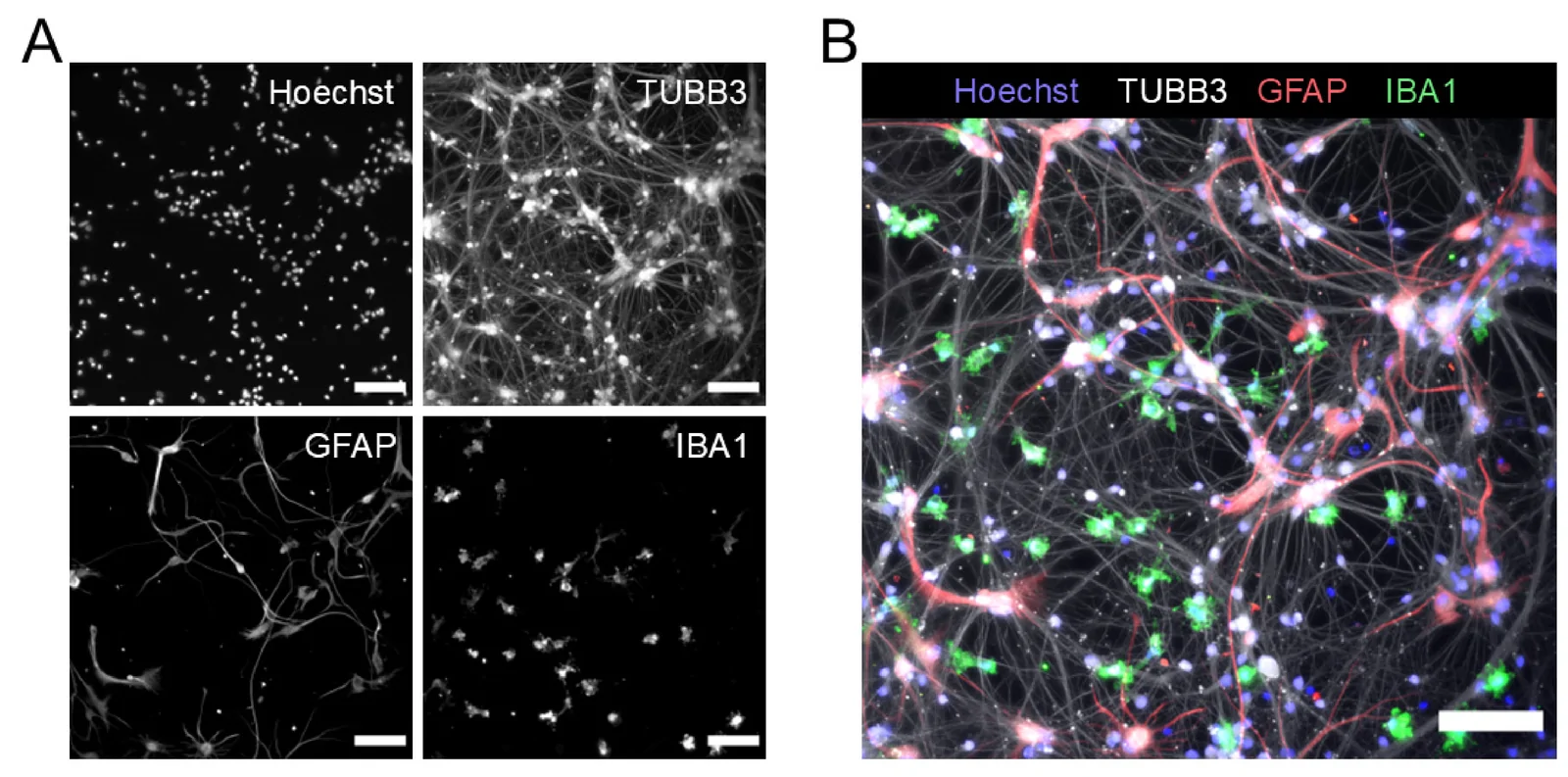
Immunofluorescence image of the hiPSC tri-culture model. The culture was fixed and tested for biomarkers TUBB3 (neurons, white), GFAP (astrocytes, red) and IBA1 (microglia, green) at the end of the experiment (day in vitro 32). Nuclei are stained with Hoechst (blue). Shown are representative (**A**) single images and (**B**) a composite image. Scale bar = 100 μm.

**Figure 2:**
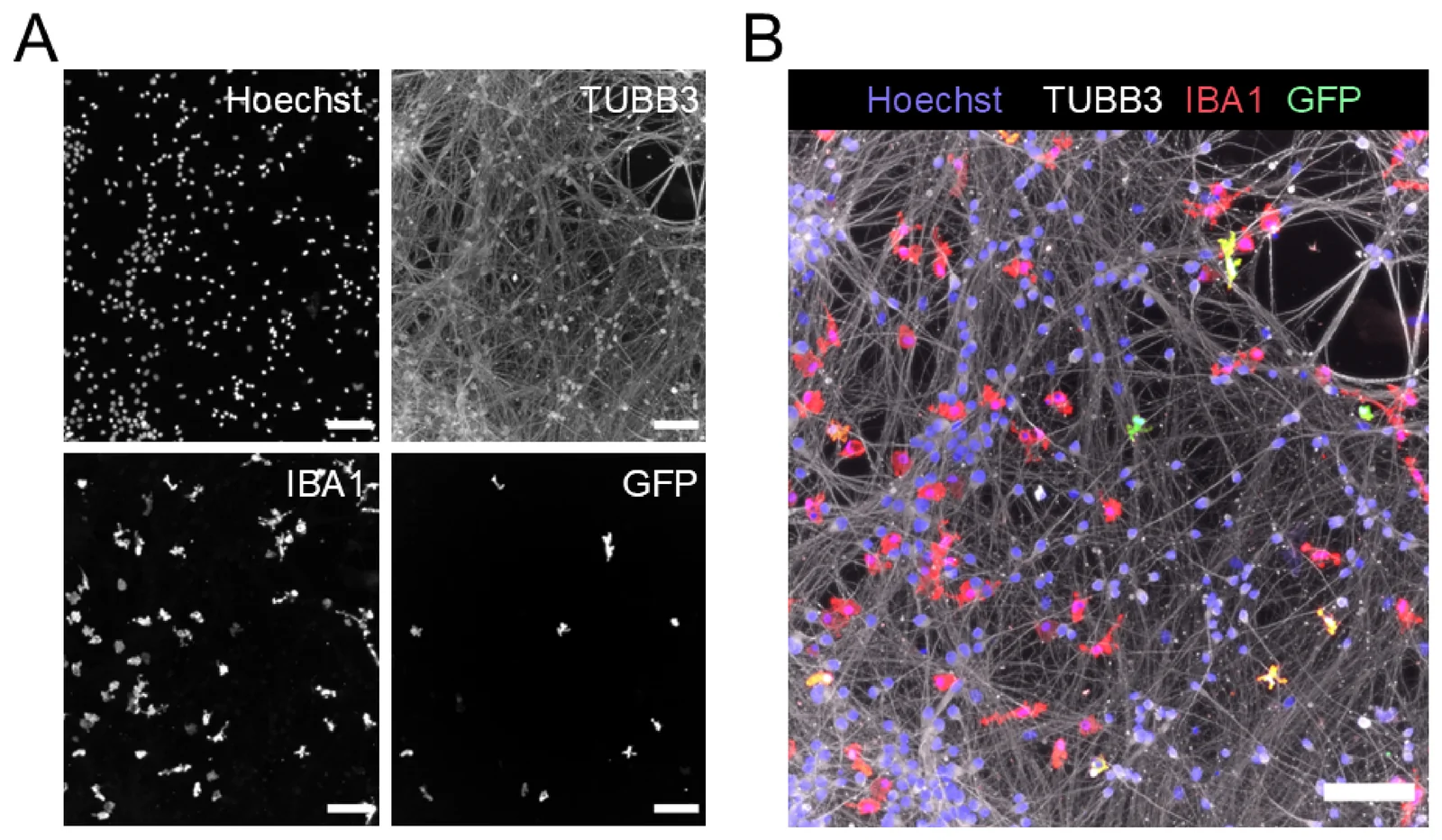
HIV-infected tri-cultures were tested for biomarkers TUBB3 (neurons, white), and IBA1 (microglia, red) at the end of the experiment. HIV is represented by GFP-expression (green) and nuclei are stained with Hoechst (blue). Shown are representative (**A**) single images and (**B**) a composite image. Scale bar = 100 μm. All detectable GFP-positive cells were IBA1-positive; no GFP signal was detected in TUBB3-positive neurons.

**Figure 3:**
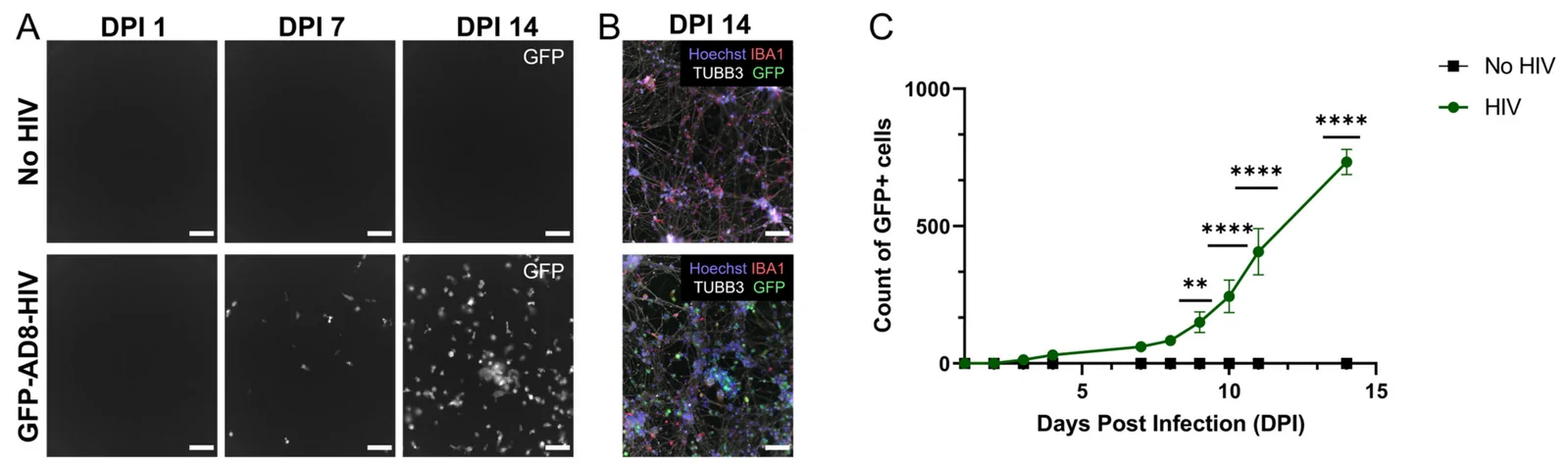
HIV replication can be tracked in the hiPSC-NeuroHIV tri-culture system. (**A**) Images from day post-infection (DPI) 1, 7, and 14 of tri-culture infected with 1:250 dilution of GFP-HIV-AD8 or uninfected control (no HIV) to demonstrate spread of HIV infection over time. Scale bar = 100 μm. (**B**) At DPI 14, cultures were fixed and labeled for neurons (TUBB3, white), microglia (IBA1, red), and nuclei (Hoechst, blue). GFP+ microglia (green) are still visible after fixation. Scale bar = 100 μm. (**C**) Shows the count of GFP+ microglia at each day of imaging in the tri-culture. Each day on the plot shows mean ± SD (N = 6). Statistical analysis was performed using repeated-measures two-way ANOVA (** *p* = 0.0053, **** *p* < 0.0001, Šídák’s multiple-comparisons test).

**Figure 4:**
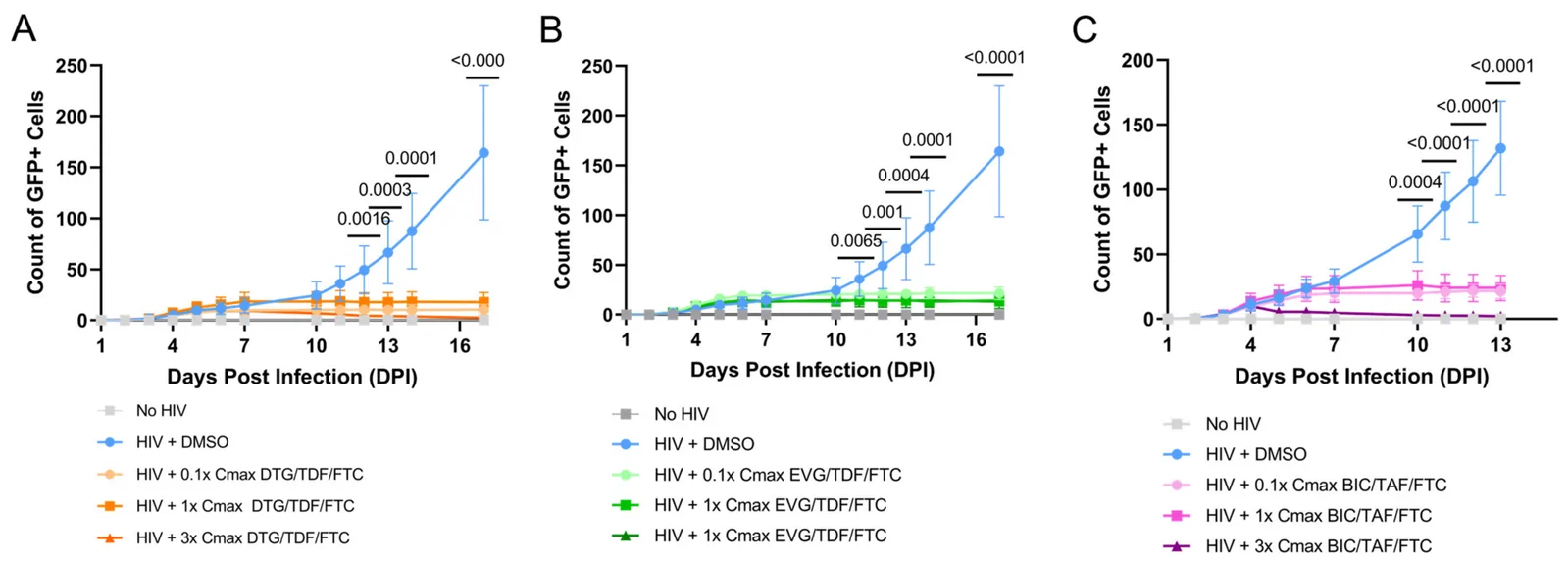
ARV combinations inhibit HIV replication in hiPSC-NeuroHIV tri-culture. The number of GFP+ microglia was quantified over the course of the experiment and plotted to assess viral replication in microglia following treatment with DTG/TDF/FTC (**A**), EVG/TDF/FTC (**B**), or BIC/TAF/FTC (**C**). Data are presented as mean ± SD of the number of GFP+ microglia per well (N = 6 wells). Statistical analysis was performed using repeated-measures two-way ANOVA followed by Šídák’s multiple-comparisons test. Exact adjusted *p*-values from Šídák’s multiple comparisons between untreated HIV-infected and HIV-infected cultures treated with 1× Cmax are shown in the graphs. DTG, dolutegravir; TDF, tenofovir disoproxil fumarate; FTC, emtricitabine; EVG, elvitegravir; TAF, tenofovir alafenamide; BIC, bictegravir.

**Figure 5:**
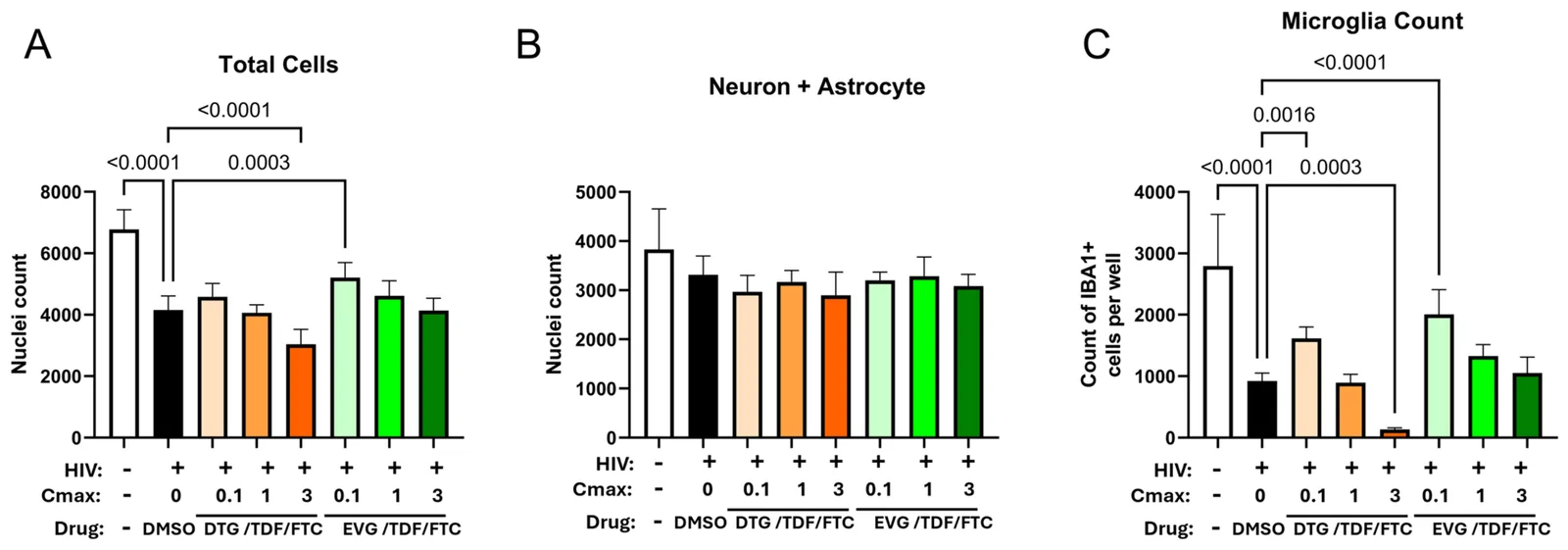
HIV infection reduces survival of microglial cells in tri-cultures. Fixed images of microglia labeled with IBA1, neurons labeled with TUBB3, and all nuclei labeled with Hoechst were used to quantify the total number of cells (**A**), neurons and astrocytes (IBA1− nuclei) (**B**), as well as microglia (IBA1+ nuclei) (**C**), remaining at experimental day 28 (DIV 28). Data are presented as mean ± SD (N = 6 wells). Statistical analysis was performed using one-way ANOVA followed by Dunnett’s multiple-comparisons test. Exact adjusted *p*-values for comparisons between untreated HIV-infected cultures and each treatment group are shown in the graphs. DTG, dolutegravir; TDF, tenofovir disoproxil fumarate; FTC, emtricitabine; EVG, elvitegravir.

**Figure 6:**
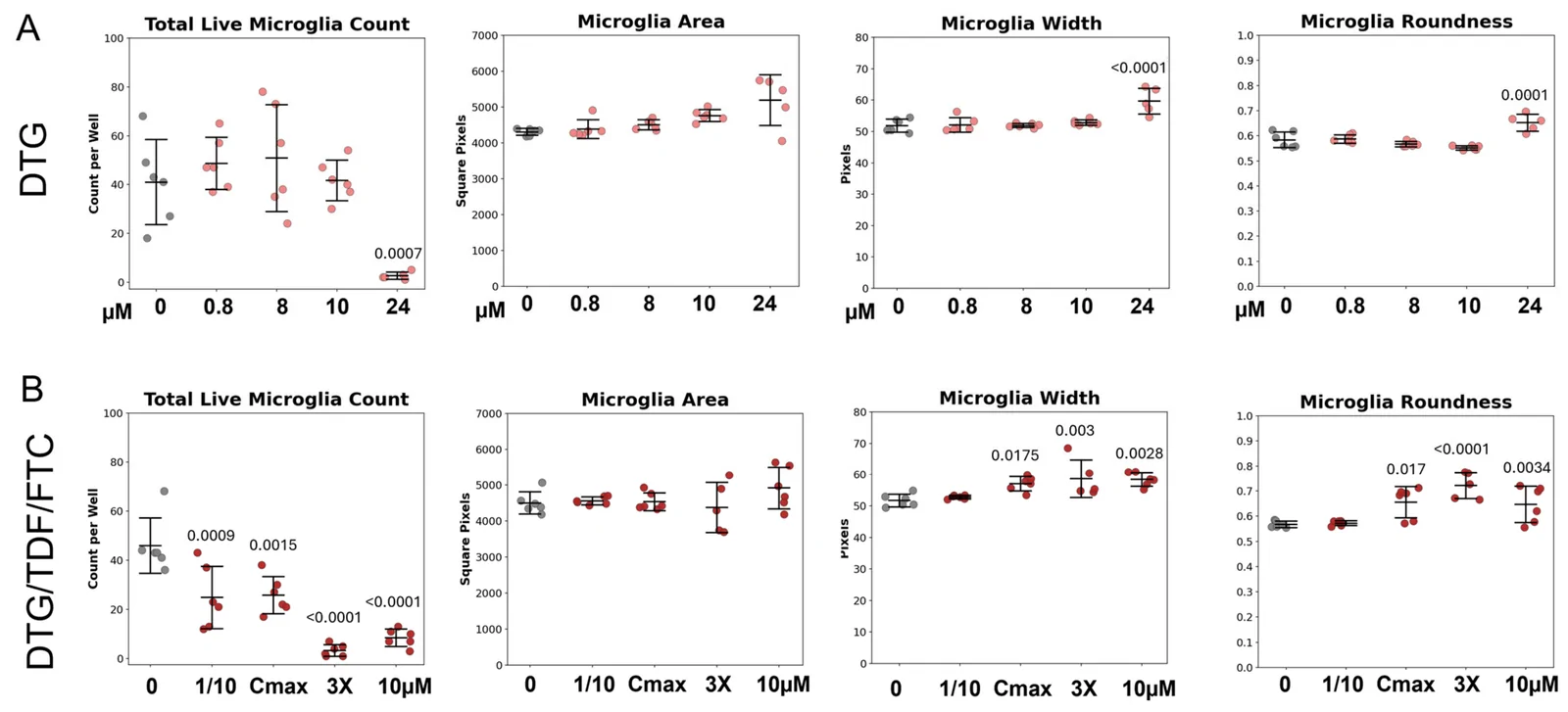
Dolutegravir and combination dolutegravir-containing ARVs reduce microglia count and alter morphology. Single (pink) or combination (red) ARVs and DMSO (control, grey) were added to tri-cultures of neurons, astrocytes, and microglia for 7 days at 0.1× Cmax, Cmax, 3× Cmax or 10µM for each compound. Data are shown as means ± SD (N = 6 wells). Statistical analysis was performed using one-way ANOVA followed by Dunnett’s multiple-comparisons test. Exact adjusted *p*-values for comparisons between DMSO (control) cultures and each treatment group are shown in the graphs. DTG, dolutegravir; TDF; tenofovir disoproxil fumarate; FTC.

**Figure 7:**
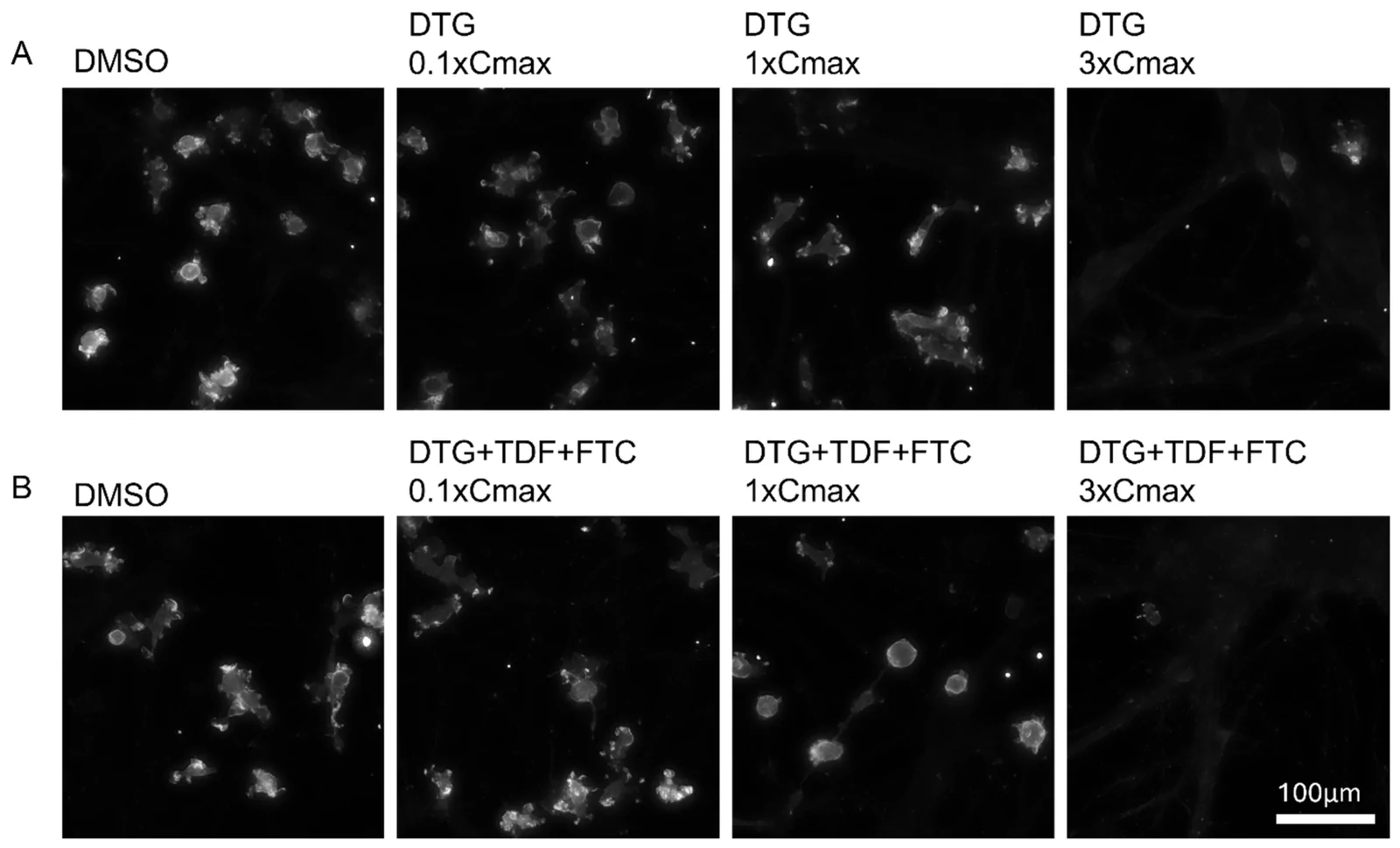
Measuring microglia morphology changes after ARV treatment of Tri-cultures. IBA1 (gray) images are shown for DMSO control and treatment with (**A**) DTG or (**B**) DTG/TDF/FTC at 0.1× Cmax, 1× Cmax, and 3× Cmax for 7 days. DTG, Dolutegravir; TDF, Tenofovir Disoproxil; Fumarate, FTC; emtricitabine. Scalebar: 100 µm.

**Figure 8:**
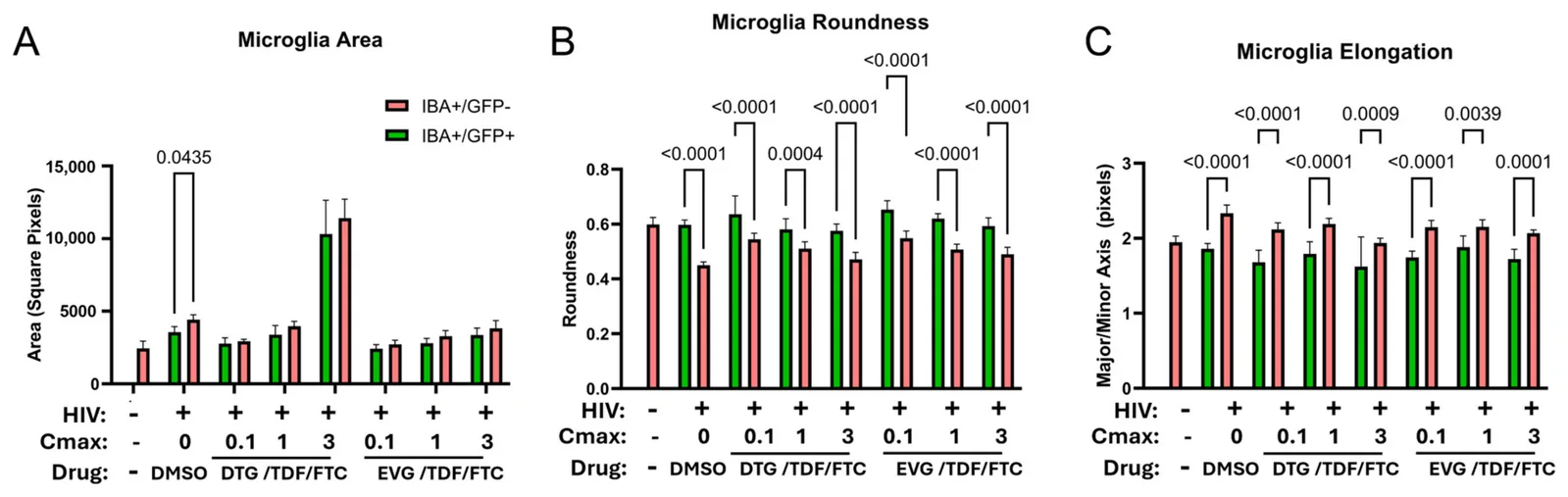
HIV infection alters morphology of microglial cells in tri-cultures. Fixed images of microglia labeled with IBA1, neurons labeled with TUBB3, and all nuclei labeled with Hoechst were used to assess changes in microglia morphology on Experiment D28, quantifying microglia area (**A**), microglia roundness (**B**), and microglia elongation (**C**) of HIV-infected and non-infected cells. Measurements of microglia morphology are shown for each treatment condition as either infected (IBA+, GFP+ (green)) or non-infected (IBA+, GFP- (pink)) microglia. Data are shown as mean ± SD (N = 6 wells). Statistical analysis was performed using two-way ANOVA followed by Šídák’s multiple-comparisons test. Exact adjusted *p*-values from Šídák’s multiple comparisons between HIV-infected cultures and HIV-non-infected cultures are shown in the graphs. DTG, dolutegravir; TDF, tenofovir disoproxil fumarate; FTC, emtricitabine; EVG, elvitegravir.

**Figure 9:**
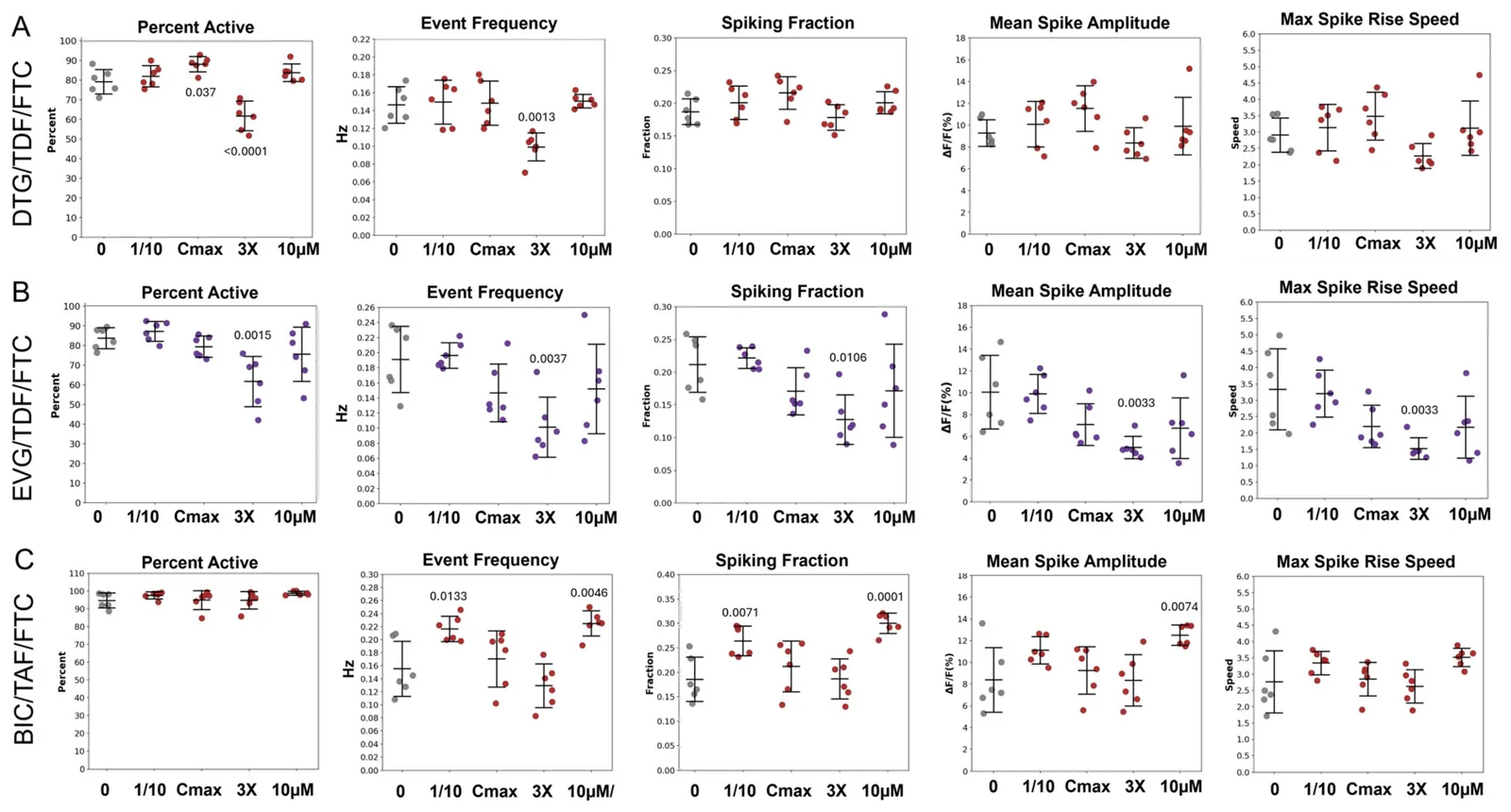
Combination ARVs show altered calcium activity of neurons in tri-cultures. Combination ARVs (DTG/TDF/FTC (red), EVG/TDF/FTC (purple), or BIC/TAF/FTC (red)) or control (DMSO, grey) were added to tri-cultures of neurons, astrocytes and microglia for 7 days at 0.1× Cmax, Cmax, 3× Cmax and 10µM. Data are shown as means ± SD (N = 6 wells). Statistical analysis was performed using one-way ANOVA followed by Dunnett’s multiple-comparisons test. Exact adjusted *p*-values for comparisons between DMSO (control) cultures and each treatment group are shown in the graphs. DTG, Dolutegravir; TDF, tenofovir disoproxil fumarate; FTC, emtricitabine; TAF, tenofovir alafenamide; BIC, bictegravir.

**Figure 10:**
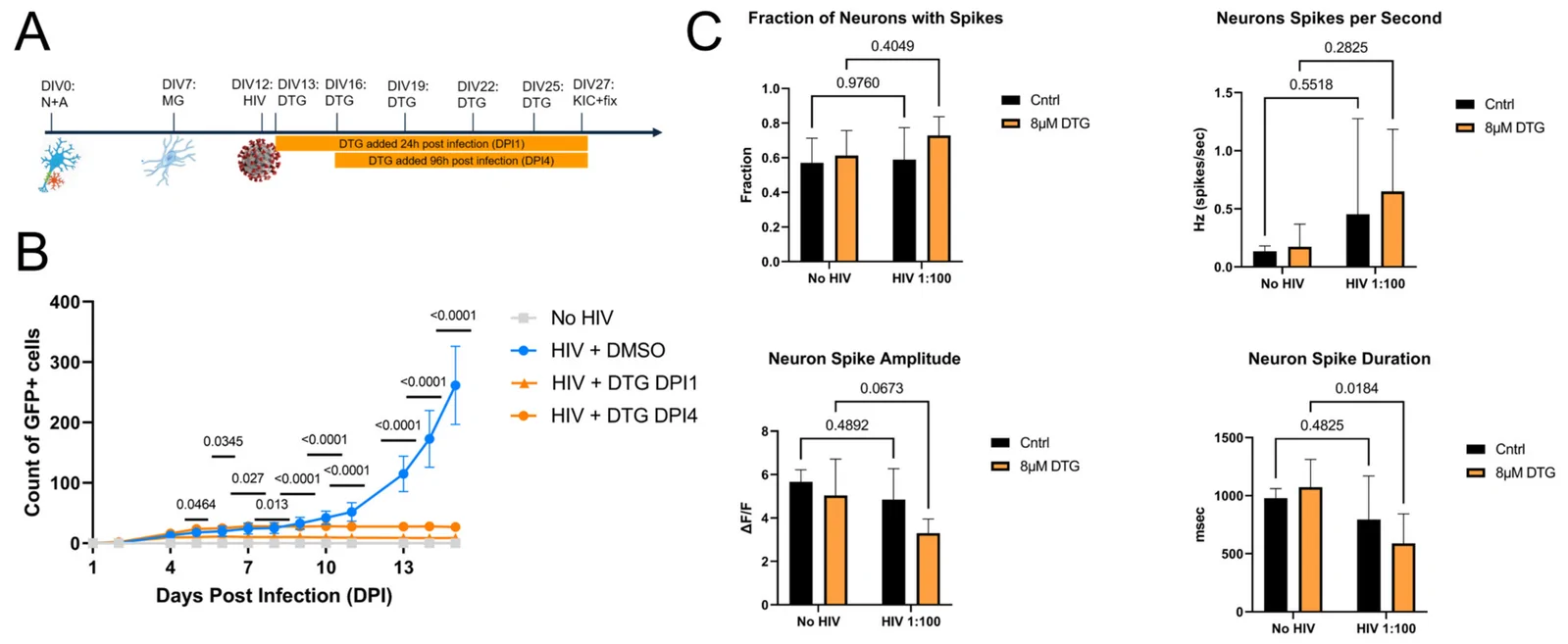
HIV replication can be detected in the tri-culture and HIV+DTG shortens neuronal spike duration. Experimental timeline is shown in (**A**) with two DTG treatment schedules (DTG was added at DPI 1 or DPI 4). (**B**) GFP+ microglia were assessed from imaging the plate throughout the experimental time course for each condition. Data are presented as mean ± SD of the number of GFP+ microglia per well (N = 6 wells). Statistical analysis was performed using repeated-measures two-way ANOVA followed by Šídák’s multiple-comparisons test. Exact adjusted *p*-values from Šídák’s multiple comparisons between untreated HIV-infected cultures and HIV-infected cultures treated with DTG at DPI 1 are shown in the graphs. (**C**) Several neuronal calcium measurements are shown after excluding microglia from the analysis. Data are from cultures treated with DTG at DPI 1. Bar graphs show the mean value for each well ± SD (N = 6). Statistical analysis was performed using two-way ANOVA followed by Šídák’s multiple-comparisons test. Exact adjusted *p*-values from Šídák’s multiple comparisons between HIV-infected and HIV-non-infected cultures within each treatment condition are shown in the graphs.

## Data Availability

The data that support the findings of this study are available from the corresponding author upon reasonable request. A formal data-sharing agreement and non-disclosure agreement may be required to protect the proprietary interests of Vala Sciences, Inc.

## Abbreviations

The following abbreviations are used in this manuscript:

ARV	Anti-retroviral
BIC/TAF/FTC	bictegravir/tenofovir alafenamide/emtricitabine
Cmax	Maximum concentration of a drug in the blood
CNS	central nervous system
DIV	Day in vitro
DPI	Day post infection
DTG/TDF/FTC	dolutegravir/tenofovir disoproxil fumarate/emtricitabine
EVG/TDF/FTC	elvitegravir/tenofovir disoproxil fumarate/emtricitabine
GFP	Green fluorescent protein
HAND	HIV-associated neurocognitive disorders
hiPSC	human induced pluripotent stem cell
INSTI	integrase strand transfer inhibitor
NRTI	nucleoside reverse transcriptase inhibitor
PWH	People with HIV
PWoH	People without HIV
